# Efficacy of Deferoxamine in Animal Models of Intracerebral Hemorrhage: A Systematic Review and Stratified Meta-Analysis

**DOI:** 10.1371/journal.pone.0127256

**Published:** 2015-05-22

**Authors:** Han-Jin Cui, Hao-yu He, A-Li Yang, Hua-Jun Zhou, Cong Wang, Jie-Kun Luo, Yuan Lin, Tao Tang

**Affiliations:** 1 Institute of Integrative Medicine, Xiangya Hospital, Central South University, Changsha, 410008, Hunan, China; 2 Institute of Mental Health, The Second Xiangya Hospital, Central South University, Changsha, 410011, Hunan, China; 3 Institute of Neurology, Xiangya Hospital, Central South University, Changsha, 410008, Hunan, China; 4 Key Lab of Chinese Gan of SATCM, Changsha, 410008, Hunan, China; 5 Institute of Neurology, The First College of Clinical Medical Sciences, China Three Gorges University, Yichang, 443003, Hubei, China; St Michael's Hospital, University of Toronto, CANADA

## Abstract

Intracerebral hemorrhage (ICH) is a subtype of stroke associated with high morbidity and mortality rates. No proven treatments are available for this condition. Iron-mediated free radical injury is associated with secondary damage following ICH. Deferoxamine (DFX), a ferric-iron chelator, is a candidate drug for the treatment of ICH. We performed a systematic review of studies involving the administration of DFX following ICH. In total, 20 studies were identified that described the efficacy of DFX in animal models of ICH and assessed changes in the brain water content, neurobehavioral score, or both. DFX reduced the brain water content by 85.7% in animal models of ICH (-0.86, 95% CI: -.48- -0.23; P < 0.01; 23 comparisons), and improved the neurobehavioral score by -1.08 (95% CI: -1.23- -0.92; P < 0.01; 62 comparisons). DFX was most efficacious when administered 2–4 h after ICH at a dose of 10–50 mg/kg depending on species, and this beneficial effect remained for up to 24 h postinjury. The efficacy was higher with phenobarbital anesthesia, intramuscular injection, and lysed erythrocyte infusion, and in Fischer 344 rats or aged animals. Overall, although DFX was found to be effective in experimental ICH, additional confirmation is needed due to possible publication bias, poor study quality, and the limited number of studies conducting clinical trials.

## Introduction

Intracerebral hemorrhage (ICH) is associated with ~15% of all strokes and exhibits high morbidity and mortality[1]. Treatments evaluated to date have shown limited efficacy and utility, and no treatment with clinically proven effectiveness has yet been identified[2].Previous studies have shown that erythrocyte rupture in the brain of animals with ICH induces an approximately three-fold increase in the nonheme iron level within the brain, and that this level remains high for at least one month[3,4]. Nonheme iron catalyzes free radical formation, which is the critical hub in the toxic cascade causing brain edema, neuronal death, brain atrophy, and poor neurologic outcomes after ICH[4–6]. Clinical studies have shown that an increased level of serum ferritin after ICH is closely related to exacerbation of brain edema and poor patient outcomes[7,8]. Chelated ferric iron and hemosiderin can form a stable complex with iron chelators, preventing iron from entering the Haber–Weiss reaction[9]. Thus, the removal of excess iron using iron chelators is a common practice. As a potent iron chelator, deferoxamine (DFX) has great potential to prevent poststroke injury caused by iron overload and iron-mediated toxicity[10]. DFX exhibits various neuroprotective effects, including inhibition of apoptosis, oxidative stress, phagocytosis, and inflammation[11]. As a promising neuroprotective drug, DFX has been repeatedly tested in several *in vivo* animal models of ICH. Positive results of DFX treatment have been reported, including reductions in iron accumulation and brain edema, as well as improvements in neurologic outcomes[11–13]. Wu et al[14]. found that DFX treatment reduced neuronal loss and improved neurologic function, but did not reduce brain injury volume, edema, or swelling in ICH mice. Additionally, Warkentin et al[2] failed to demonstrate beneficial therapeutic effects of DFX. Moreover, the results of many drug studies involving animals are discrepant from those of human clinical studies[15,16], possibly due to differences in treatment time windows. Thus, any potential clinical trial strategies should rely on a comprehensive and unbiased systematic evaluation of animal data and a consideration of their limitations. This review examines the impact of study quality and various study characteristics on effect size to determine whether the currently available evidence from animal experiments supports the therapeutic use of DFX for ICH.

## Materials and Methods

### Data sources, search strategy, and selection criteria

The following online databases were searched for relevant studies published between 2002 and September 2014: PubMed, Web of Knowledge, Embase, China National Knowledge Infrastructure, VIP Database for Chinese Technical Periodicals, Wanfang Database, and Chinese Biomedical Literature Database. The following search terms were used: intracerebral h(a)emorrhage OR ICH OR intracranial h(a)emorrhage OR h(a)emorrhagic stroke OR stroke AND deferoxamine OR DFX OR desferrin OR Desferal OR desferrioxamine OR deferoxaminum OR deferoxamine mesylate OR desferrioxamine B mesylate OR DFX OR DFM OR DFOM OR DFO OR Ba-33112, NOT human OR patient. The reference lists of all included studies were searched as well. Studies were included if they fulfilled the following criteria: (1) experimental ICH was induced and the therapeutic effect of DFX was assessed; (2) control animals were used; (3) DFX was administered after the induction of ICH; (4) no cotreatments were performed; and (5) effect of DFX was assessed by brain water content or neurobehavioral outcome, as brain iron concentrations can reach 10 mmol/L after ICH, resulting in severe brain edema[17], which is the most life-threatening and devastating complication of ICH[18], and brain edema surrounding the hematoma has been shown to be closely related to poor outcome[19,20]. Two reviewers (Cui HJ and He HY) independently screened the abstracts according to the inclusion criteria, and disagreements were addressed by discussion with a third reviewer (Tang T).

### Data extraction

The following data were extracted from the included studies: methodological quality score; animal species; number, sex, and age of animals studied; time, route, and dose of drug administration; ICH induction method; anesthetic technique used during the operation; efficacy assessment methods; whether random and blind strategies were used; and treatment outcomes.We extracted data regarding the number of animals per group and outcome parameters (mean and standard deviation) from both the control and treatment groups to compare the drug efficacy. When dose-response relationships were assessed within multiple groups, the data from each group were extracted individually for analysis. Scientific graphing and data analysis software (OriginPro 9.0; OriginLab Corporation, Northampton, MA, USA) was used to measure graphically presented data. When data were expressed serially at different time points (e.g., neurologic tests), the final time point was extracted and only the result of the final test was included. When it was unclear whether the measure of variance was the standard deviation or standard error of the mean, we extracted the data as the standard error of the mean because this was a more conservative estimate for the purpose of the present meta-analysis. When a single group of animals underwent assessment of more than one neurologic aspect (e.g., motor and sensory scores), the data were combined to obtain an overall estimate of the magnitude of the effect and standard error. Two reviewers (Zhou HJ and Yang AL) independently extracted the data.

### Quality assessment

We used the Stroke Therapy Academic Industry Roundtable (1999) rating system to assess the methodological quality of each study[21]. This rating system has been validated, and is commonly used for assessing the quality of animal studies[22–24]. One point was given for each of the following criteria: presence of randomization, assessment of dose-response relationship, assessment of optimal time window, monitoring of physiologic parameters, blinded outcome assessment, assessment of at least two outcomes, acute-phase outcome assessment (1–3 days), and chronic-phase outcome assessment (7–30 days). Studies that scored < 4 points were considered to be of poor methodological quality, and studies that scored ≥ 4 points were considered to be of good methodological quality.

### Data analysis

The data were analyzed using a statistical software package (Stata, version 11.0; StataCorp LP, College Station, TX, USA). The effect of DFX on the total brain water content and the neurologic outcomes were compared between the treatment and control groups using the standardized mean difference (the difference in the effect of DFX between the treatment and control groups was divided by the total standard deviation). We used the DerSimonian and Laird random-effects model to pool these estimates[25]. This model is not only more conservative than a fixed-effects model, but also takes into consideration any statistical heterogeneity found between studies.A stratified meta-analysis was performed to examine the impact of drug dose, time of administration, overall study quality score, method of ICH induction, species and age of animals used, and type of anesthetic used. Publication bias was detected by funnel plotting; asymmetry was assessed using an Egger’s test and the trim-and-fill method[26]. Statistical significance was set at *P* < 0.05, and the 95% confidence intervals (CIs) of all results were calculated.

### Assessment and exploration of heterogeneity

In the evaluation of heterogeneity, Q was the heterogeneity statistic and *df* reflected the percentage of variability caused by heterogeneity rather than by sampling error among studies.

## Results

**Fig 1:**
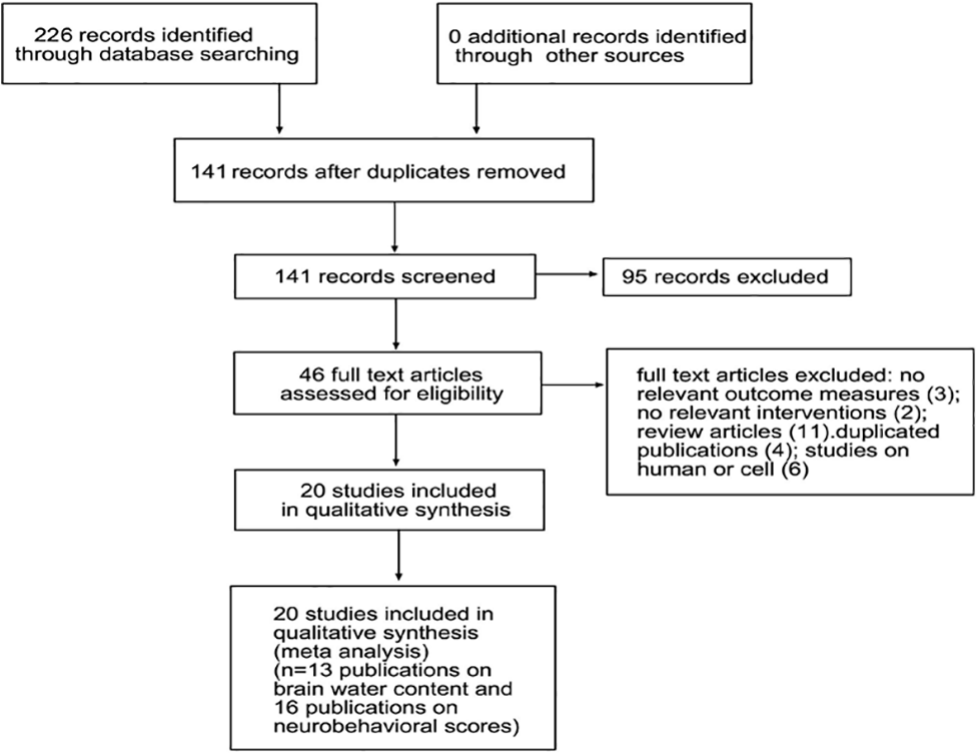
Progression from literature search to meta-analysis. The number of exclusions from the initial literature search is shown.

We identified 226 publications from the above-described electronic search; 180 were excluded due to duplication (*n* = 85) and failure to meet the inclusion criteria (*n* = 95). We screened 46 publications in detail and excluded an additional 26 publications because of a lack of relevant outcome measures (*n* = 3), a lack of relevant interventions (*n* = 2), the performance of a review only (*n* = 11), duplicated publication (*n* = 4), and the use of humans or cells as the study subjects (*n* = 6) (Fig 1).Finally, this systematic review included 20 articles published between 2002 and 2014 that met the inclusion criteria. We extracted the data from 86 comparisons describing the brain water content among 13 studies, as well as the data on the neurobehavioral scores among 16 studies (Fig 1). The 20 included studies involved rats (*n* = 18), mice (*n* = 1)[14], and pigs (*n* = 1)[27]. Most studies used male animals (*n* = 19); one publication used both male and female animals[28]. Adult and aged animals were most commonly used; one publication used young animals[27], and five publications did not report the age of the animals.The overall study characteristics are shown in Table 1. Drugs were administered via intraperitoneal injection in the majority of studies (*n* = 15), and by intramuscular injection in most of the remaining studies (*n* = 4); one study did not describe the route of administration [29]. The timing of drug administration ranged from 0 to 72 h after the induction of ICH. In 36/86 (42%) unique comparisons of animals, the drug was administered 2 h after ICH induction; in 19/86 (22%) comparisons, the drug was administered 6 h after ICH induction. Assessment was performed 24 h to 56 d after induction of ICH.

**Table 1:** Design characteristics of included studies.

Author, yr	Animal(DFX/Con),n	Sex	Age	Method of ICH	Anesthetic	Intervention	Assessment	R	B	Q	C
Huang et al., 2002[17]	SDR (5/5)	M	NR	Hemoglobin30 μl/CN	phB, i.p.	50, 500 mg/kg, i.p. started immediately after ICH	BWC (24 h after ICH)		√	5	+
Jin [40] (unpublished Master thesis, 2004)	SDR (12/12)	M	N	Whole blood100 μl/CN	PS, i.p.	100 mg/kg every 12 h i.p., started 24 or 72 h after ICH for 3 days	NS, BWC (1, 2, 3, 7, 14, and 21 d)	√	√	7	+
Nakamura et al., 2003[11]	SDR (36/36)	M	NR	Whole blood100 μl/BG	phB, i.p.	100 mg/kg every 12 h i.p., started 2, 6, or 24 h after ICH until the animals were killed	BWC (3 d after ICH), NS (1, 3, and 7 d after ICH)			5	+
Wu et al., 2005[29]	SDR (6/6)	M	NR	Lysed erythrocytes 30 μl/CN	phB, i.p.	150 mg/kg i.p. (time window unknown)	NS, BWC (24 h after ICH)	√		4	+
Hua et al., 2006[41]	SDR (6/6)	M	NR	Whole blood100 μl/CN	PS, i.p.	100 mg/kg every 12 h i.p., started 2 h after ICH for 7 days	NS (2 and 4 weeks after ICH)	√	√	5	+
Wan et al., 2006[3]	SDR (6/6)	M	NR	Whole blood100 μl/BG	phB, i.p.	100 mg/kg at 12-h intervals i.p., started 2 h after ICH for 7 days	NS (1, 3, 7, 14, 21, and 28 d after ICH)		√	5	+
Bao et al., 2008[42]	SDR (5/5)	M	NR	Hemoglobin30 μl/CN	phB, i.p.	100 mg/kg i.p., started immediately after ICH	BWC (24 h after ICH)	√		4	+
Liu et al., 2008[43]	SDR (11/11)	M	N	Whole blood50 μl/CN	CH, i.p.	100 mg/kg every 12 h i.p., started 24 h after ICH until the animals were killed	NS, BWC (1, 3, 5, and 7 d after ICH)	√		4	+
Okauchi et al., 2009[13]	FR (8/8)	M	A	Whole blood100 μl/BG	phB, i.p.	10, 50, and 100 mg/kg every 12 h i.m., started 2 h after ICH for 3 or 7 days	NS (1, 28, and 56 d after ICH), BWC (3 d after ICH)	√	√	8	+
Wan et al., 2009[44]	SDR (10/10)	M	N	Whole blood50 μl/BG	phB, i.p.	100 mg/kg every 12 h i.p., started 2 h after ICH	NS (1, 3, 7, 14, and 28 d)	√	√	6	+
Wang et al., 2009[34]	SDR (9/9)	M/F	NR	Whole blood100 μl/BG	CH, i.p.	50 mg every 12 h i.p., started 24 h after ICH	BWC (1, 3, 7, and 14 d)	√		4	+
Qiu[45] (unpublished Masters thesis, 2010)	SDR (6/6)	M	NR	Whole blood100 μl/CN	CH, i.p.	100 mg/kg every 12 h i.p., started 2 h after ICH for 7 days	NS (1, 3, 7, 14, and 28 d after ICH)	√	√	6	+
Warkentin et al., 2010[2]	SDR (48/24)	M	N	Collagenase VII0.075 U/Str	Iso, Inhal.	100 mg/kg every 12 h i.p., started immediately or 6 h after ICH for 3 or 7 days	NS (3, 7, 14, and 28 d after ICH), BWC (3 d after ICH)	√	√	7	-
Hatakeyama et al., 2011[46]	FR (18/12)	M	A	Whole blood100 μl/BG	phB, i.p.	100 mg/kg at 12-h intervals, unknown, started 2 h after ICH for 7 days	NS (1, 28, and 56 d after ICH)		√	6	+
Wu et al., 2005[29]	CM (10/10)	M	A	Collagenase VII0.075 U/Str	Avertin, i.p.	200 mg/kg every 12 h i.p., started 6 h after ICH for 3 days	NS (1 and 3 d), BWC 3 d after ICH	√	√	4	-
Auriat et al., 2012[47]	SDR (8/8)	M	N	Collagenase IV0.1 U/Str	Iso, Inhal.	100 mg/kg every 12 h i.p., started 6 h after ICH for 7 days	NS (1, 3, and 7 d) after ICH	√	√	6	+
Chun et al., 2012[48]	SDR (11/11)	M	N	Collagenase IV0.23 U/Str	T/Z, i.p.	50 mg/kg daily i.p. (time window unknown) for 3 or 7 days	NS (28 d), BWC (3 d)	√	√	6	+
Hatakeyama et al., 2013[49]	FR (9/9)	M	A	Whole blood100 μl/BG	phB, i.p.	100 mg/kg at 12-h intervals i.m., started 2 h after ICH for 7 days	NS (1, 3, and 7 d after ICH)		√	5	+
Okauchi et al., 2010[12]	FR (8/8)	M	A	Whole blood100 μl/BG	phB, i.p.	First set: 50 mg/kg, started 2, 4, 12, or 18 h after ICH; second injection 4 h later, then every 12 h per i.m. injection for 3 daysSecond set: 50 mg/kg, started 2 h after ICH, then every 12 h i.m. for 2, 5, 7, or 14 daysThird set: 50 mg/kg, started 2, 4, 12, 24, or 48 h after ICH with the second injection 4 h later, then every 12 h per i.m. injection for 7 days	NS (1, 28 and 56 d after ICH), BWC (3 d after ICH)			6	+
Xie et al., 2014[27]	Pigs (10/9)	M	Y	Whole blood2.5 ml/FL	Iso, Inhal	50 mg/kg i.m., started 2 h after ICH with the second injection 4 h later, and then at 12-h intervals for 3 days	BWC (3 d after ICH)			4	+

DFX, deferoxamine; Con, control; SDR, Sprague–Dawley rats; FR, Fischer 344 rats; CM, C57 mice; M, male; F, female; A, aged; N, normal adult; Y, young; CN, caudate nucleus; BG, basal ganglia; Str, striatum; FL, frontal lobe; phB, phenobarbital; PS, pentobarbital sodium; Iso, isoflurane; CH, chloral hydrate; AV, avertin; T/Z, telazol/xylazine; i.p., intraperitoneal; i.m., intramuscular; Inhal, inhaled; NS, neurological score; BWC, brain water content; NR, not reported; R, random assignment; B, blinding; Q, study quality; C, conlusion

“+” indicates a positive conclusion and “-” represents a negative conclusion.

### Global estimates of efficacy

**Fig 2:**
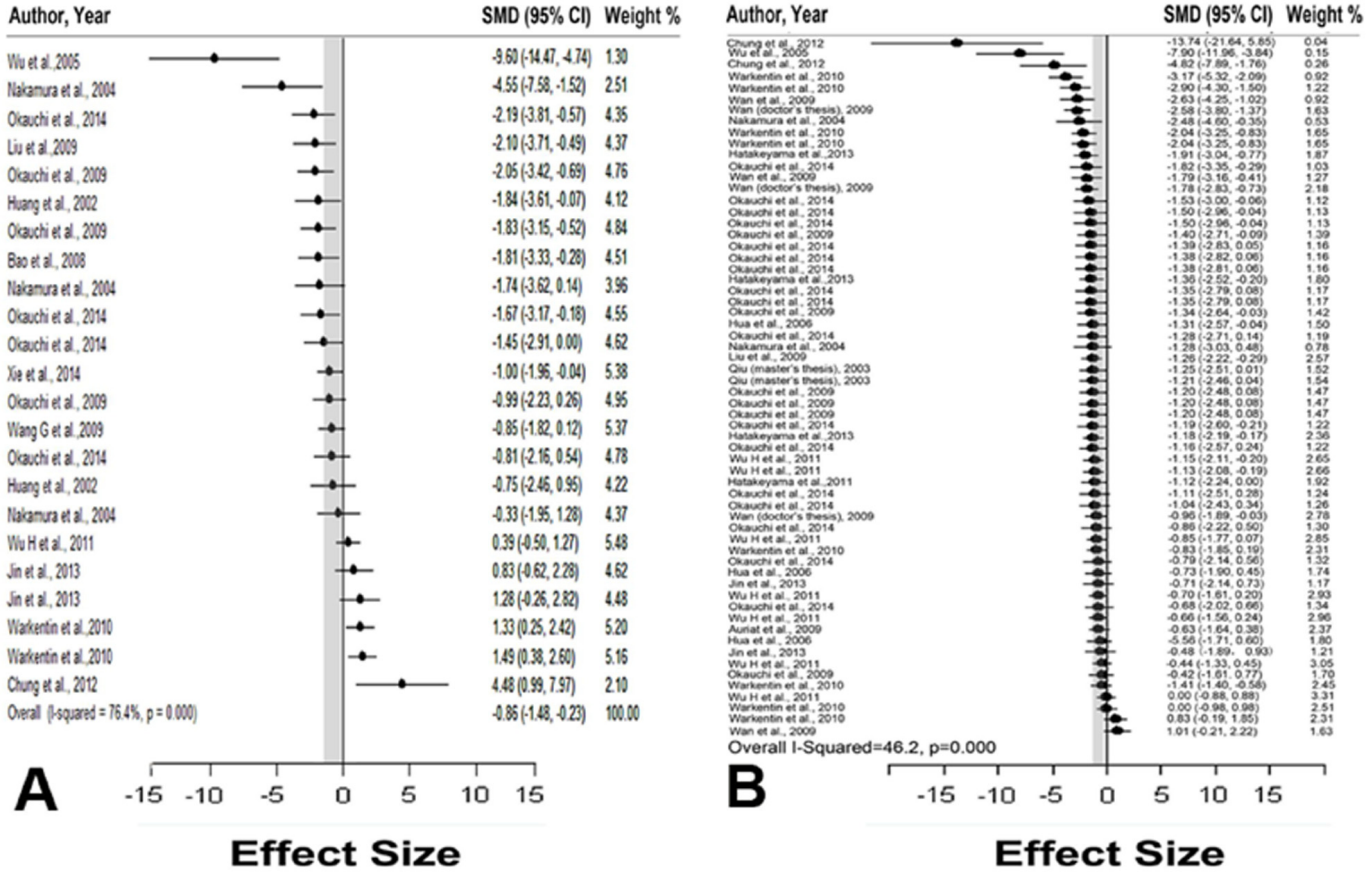
Effect sizes of included comparisons. A forest plot of the effect sizes for each comparison measuring **(A)** brain water content and **(B)** neurobehavioral outcomes. Gray bars represent 95% confidence intervals.

The global estimate of the efficacy of DFX in reducing the brain water content was—0.857 (95% CI: –1.48––0.23; 23 comparisons) (Fig 2A). Statistically significant heterogeneity was present between comparisons (*χ* ^*2*^ = 93.15, *df* = 22; *P* < 0.0001). The global estimate of the efficacy of DFX in improving the neurobehavioral outcome was—1.08 (95% CI: –1.23––0.92; 62 comparisons) (Fig 2B). The heterogeneity of neurobehavioral outcomes among studies was not statistically significant; therefore, further analysis was not performed (*χ* ^*2*^ = 113.33, *df* = 61, *P* < 0.0001).

### Publication bias

**Fig 3:**
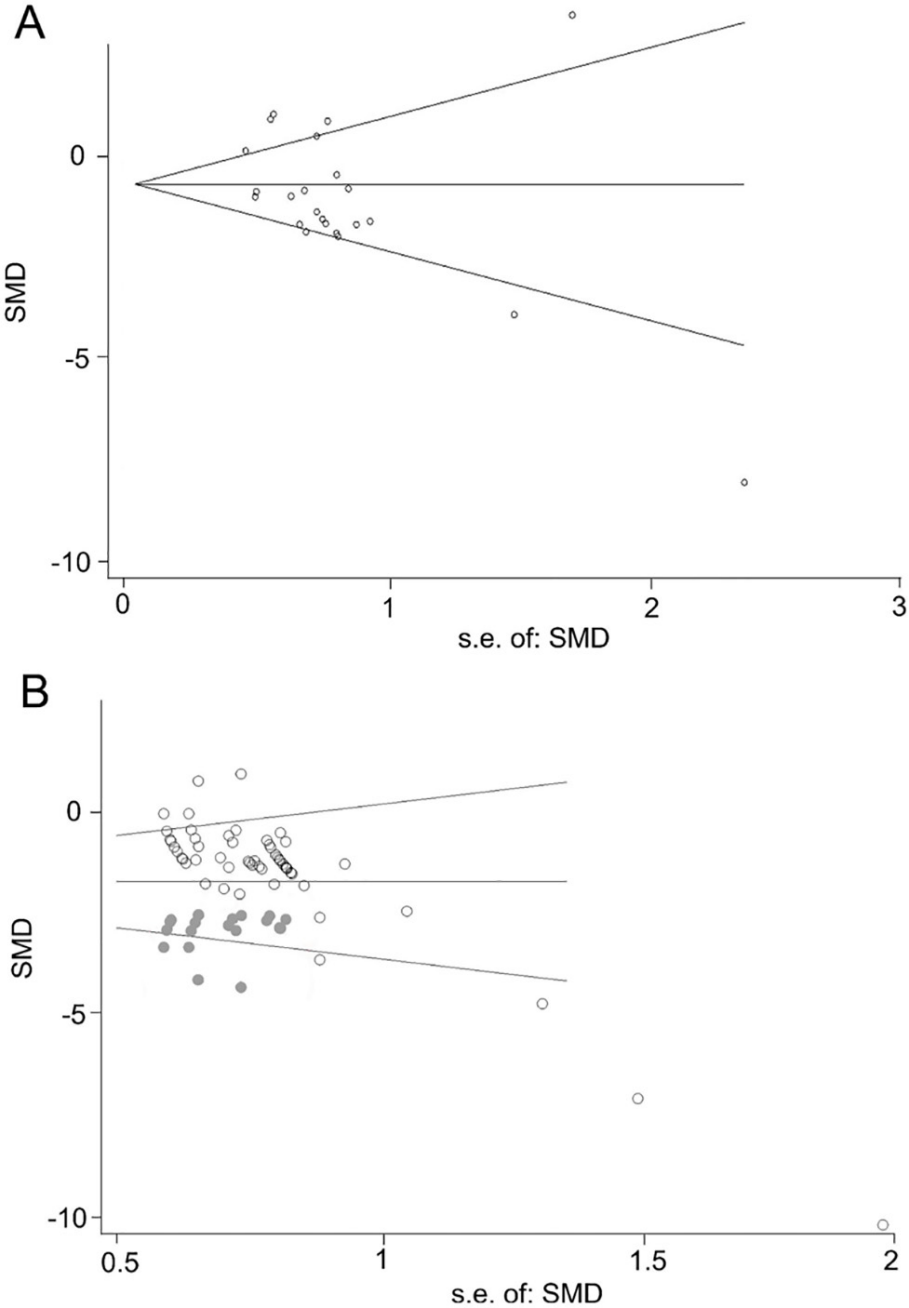
Publication bias. **(A, B)** Funnel plots. Gray points in (B) represent theoretically missing comparisons identified using the trim-and-fill method.

Visual inspection of the funnel plots indicated substantial publication bias for both the brain water content and neurobehavioral outcomes; the presence of publication bias was supported by the results of Egger’s regression (Fig 3). No theoretically missing studies that measured the brain water content were predicted by the trim-and-fill method (Fig 3A), but 17 theoretically missing comparisons of neurobehavioral outcomes were predicted (Fig 3B).

### Study quality

**Fig 4:**
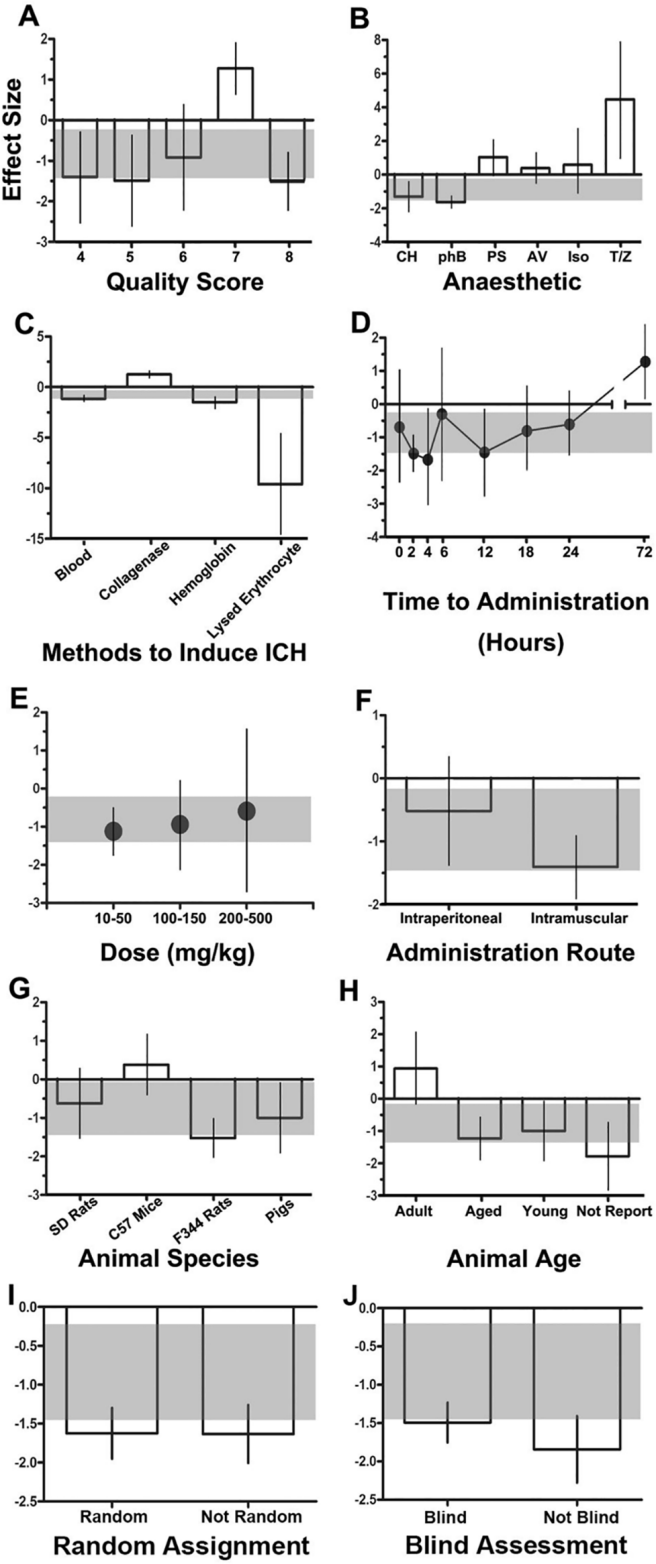
Impact of study design characteristics. This figure shows the effects of the following factors on efficacy, measured as the reduction in brain water content: **(A)** quality score, **(B)** anesthetic used during the induction of intracerebral hemorrhage (ICH), **(C)** route of deferoxamine administration, **(D)** time of administration, **(E)** drug dose, **(F)** methods of ICH induction, **(G)** animal species, **(H)** animal age, **(I)** whether random assignment was used, and **(J)** whether blind assessment was used. Error bars represent 95% confidence intervals. The horizontal gray bar represents the global estimate of efficacy for brain water content and its 95% confidence interval.

Of the 20 publications included in the systematic review, 3 (15%) investigated the dose-response relationship[12,13,17], 14 (70%) reported random allocation of animals to treatment groups, 6 (30%) investigated the optimal time window of the treatment, 18 (90%) monitored the animals’ physiologic parameters during the induction of ICH, 15 (75%) blinded the outcome assessment, 20 (100%) assessed at least two acute-phase outcomes, and 17 (85%) assessed chronic-phase outcomes (Table 2).The sample sizes were small. For evaluation of brain water content, the median numbers (interquartile range) of animals in the control and treatment groups were 9 (3.5) and 3 (2), respectively. For evaluation of neurobehavioral outcomes, the median numbers of animals in the control and treatment groups were 5 (3) and 9 (1), respectively. Overall, the median study quality score was 5 (2). Stratification according to the overall quality score elucidated significant between-study heterogeneity in brain water content; however, no clear trend was found (Fig 4A).

**Table 2:** Quality characteristics of included studies.

Authors,year of publication	(1)	(2)	(3)	(4)	(5)	(6)	(7)	(8)	Score
Huang et al., 2002[17]	√			√	√	√	√		5
Jin[40] (unpublished Master thesis, 2004)		√	√	√	√	√	√	√	7
Nakamura et al., 2003[11]			√	√		√	√	√	5
Wu et al., 2005[29]		√		√		√	√		4
Hua et al., 2006[41]		√		√	√		√	√	5
Wan et al., 2006[3]				√	√	√	√	√	5
Bao et al., 2008[42]		√		√		√	√		4
Liu et al., 2008[43]		√				√	√	√	4
Okauchi et al., 2009[13]	√	√	√	√	√	√	√	√	8
Wan et al., 2009[44]		√		√	√	√	√	√	6
Wang et al., 2010[34]		√				√	√	√	4
Qiu[45] (unpublished Masters thesis, 2010)		√		√	√	√	√	√	6
Warkentin et al., 2010[2]		√	√	√	√	√	√	√	7
Hatakeyama et al., 2011[46]			√	√	√	√	√	√	6
Wu et al., 2005[29]		√			√	√	√		4
Auriat et al., 2012[47]		√		√	√	√	√	√	6
Chun et al., 2012[48]		√		√	√	√	√	√	6
Hatakeyama et al., 2013[49]				√	√	√	√	√	5
Okauchi et al., 2010[12]	√		√	√		√	√	√	6
Xie et al., 2014[27]				√		√	√	√	4

(1) Dose-response relationship that was investigated

(2) randomization of the experiment

(3) optimal time window of the treatment investigated

(4) monitoring of physiologic parameters

(5) blinded outcome assessment

(6) assessment of at least two outcomes

(7) acute-phase outcome assessment (1–3 days)

(8) chronic-phase outcome assessment (7–30 days).

### Study characteristics

Experiments in which phenobarbital anesthesia was used during the induction of ICH showed a higher effect size than did experiments in which other anesthetics were used (effect size, –1.64, 95% CI: –2.19––1.08; *P* < 0.0001) (Fig 4B). Our comparison of studies using different methods of ICH induction showed a significantly higher effect for lysed erythrocyte infusion (effect size, –9.60, 95% CI: –14.47––4.73; *P* < 0.0001) and a moderate effect for hemoglobin infusion (effect size, –1.49, 95% CI: –2.44––0.53; *P* < 0.002). The collagenase infusion model did not favor DFX treatment (Fig 4F). All included studies involved post-ICH administration of DFX and exhibited a significant protective effect of DFX at all administration time points preceding 72 h. DFX most effectively reduced the brain water content when administrated 2 h (effect size, –1.49, 95% CI: –2.01––0.95; *P* < 0.0001) and 4 h (effect size, –1.67, 95% CI: –3.1 ––0.18; *P* < 0.05) following ICH (Fig 4D). We also analyzed the dose-response relationship of DFX and found that the median (IQR) tested dose was 100 mg/kg (50 mg/kg) in ten cohorts in which the brain water content was measured. A significant protective effect was observed at all doses of DFX. Although DFX appeared to be the most effective when administrated at a dose of 100–150 mg/kg, this effect was not statistically significant (effect size, –0.94, 95% CI: –2.14–0.27) (Fig 4E). Intramuscular injection of DFX exhibited a higher effect (effect size, –1.41, 95% CI: –1.87––0.94; *P* < 0.0001) than did intraperitoneal injection (Fig 4C).After stratification of the data according to animal species and age, the highest effect was found in Fischer 344 rats (effect size, –1.53, 95% CI: –2.0 ––1.00; *P* < 0.0001) (Fig 4G) and the group in which the animals’ age was not reported (effect size, –1.78, 95% CI: –2.83––0.73; *P* < 0.01), followed by aged animals (effect size, –1.23, 95% CI: –1.96––0.04; *P* < 0.01) (Fig 4H).Studies that did not use blind assessment showed a higher effect size (effect size, –1.36, 95% CI: –1.94––0.78; *P* < 0.0001) than those who did. However, the results showed no significant difference between studies that did or did not use random assignment.

### Heterogeneity test

Heterogeneity was investigated following the stratification. The Q-test showed that only the heterogeneity of the following subgroups was statistically significant: 4- and 6-point studies, use of isoflurane anesthesia, use of intraperitoneal DFX injection, time windows of 0 and 6 h, doses of 100–150 and 200–500 mg, use of a whole blood-induced model, use of Sprague–Dawley rats, and use of adult and aged animals (all *P <* 0.05). Other heterogeneity Q-tests within subgroups showed no statistical significance (Table 3).

**Table 3:** Stratified meta-analysis of heterogeneity.

Stratification group		No. of studies	SMD (95% CI)	Heterogeneity test		
				*Q*	*P*	*I* ^*2*^ (%)
Quality score	4	6	–0.81 (–1.30––0.33)	23.83	0.000	79.0
	5	5	–1.36 (–2.20––0.53)	6.73	0.150	40.6
	6	5	–1.21 (1.94––0.49)	12.41	0.015	67.8
	7	4	1.28 (0.66–1.91)	0.52	0.910	0.0
	8	3	–1.60 (–2.36––0.84)	1.48	0.480	0.0
Anesthetic	CH	2	–1.18 (–2.01––0.35)	1.70	0.192	41.3
	pHB	14	–1.57 (–1.99––1.14)	20.83	0.076	37.6
	PS	2	1.04 (–0.02–2.10)	0.18	0.675	0.0
	AV	1	0.39 (–0.50–1.27)	0.00		
	Iso	3	1.46 (0.14–1.06)	14.68	0.001	86.4
	T/Z	1	4.48 (–0.99–7.79)	0.00		
Administration route	i.p.	15	–0.10 (–0.47–0.27)	70.29	0.000	80.1
	i.m.	8	–0.61 (–0.90––0.32)	4.16	0.760	0.0
Time to administration, h	0	4	–0.69 (–2.40–1.01)	15.65	0.001	80.80
	2	6	–1.49 (–2.02––0.95)	3.32	0.650	0.0
	4	1	–1.67 (–3.17––0.18)	0.00		
	6	3	–0.30 (–2.39–1.79)	13.76	0.001	85.5
	12	1	–1.45 (–2.91–0.00)	0.00		
	18	1	–0.81 (–2.16–0.55)	0.00		
	24	4	–0.61 (–1.69–0.48)	7.39	0.060	59.4
	72	1	1.28 (–0.26–2.82)	0.00		
Dose, mg	10–50	10	–1.13 (–1.55––0.71)	14.85	0.095	39.4
	100–150	11	–0.19 (–0.64–0.27)	62.49	0.000	84.0
	200–500	2	–0.06 (–0.90––0.32)	4.86	0.027	79.4
Method to induce ICH	Blood	15	–1.09 (–1.44––0.72)	29.79	0.008	53.0
	Collagenase	4	1.06 (0.49–1.64)	6.72	0.081	55.4
	Hemoglobin	3	–1.49 (–2.44–0.53)	1.04	0.600	0.0
	Lysed erythrocytes	1	–9.60 (–14.47––4.73)	0.00		
Animal species	SD rats	14	–0.20 (–0.61–0.21)	68.89	0.000	81.1
	C57 mice	1	0.39 (–0.50–1.27)	0.00		
	F344 rats	7	–1.53 (–2.05––1.00)	3.27	0.774	0.0
	Pigs	1	–1.00 (–0.90––0.32)	0.00		
Animal age	Adult	6	0.94 (0.36–1.51)	19.29	0.002	74.1
	Aged	8	–1.03 (–1.48––0.57)	16.52	0.021	57.5
	Young	1	–1.00 (–1.96––0.04)	0.00		

phB, phenobarbital; PS, pentobarbital sodium; Iso, isoflurane; CH, chloral hydrate; AV, avertin; T/Z, telazol/xylazine; i.p., intraperitoneal; i.m., intramuscular; SD, Sprague–Dawley.

## Discussion

This is the first systematic review and meta-analysis on DFX in animal models of ICH. The findings of this systematic review indicate that DFX is neuroprotective in terms of its impact on both reducing the brain water content and improving neurobehavioral outcomes in ICH models. However, the results should be interpreted with caution because of limitations including possible publication bias, poor study quality, and the limited number of studies. These limitations are discussed below.

### Study quality

Although the highest effect size of DFX was found in 8- and 5-point studies, those with lower quality scores were more likely to overstate the effect sizes. Additionally, we noticed that measurements to reduce bias, such as dose-response relationship investigation, blindness during outcome assessment, and chronic-phase assessment, were neglected in more than half of these 4-point studies.

### Study design

DFX was most efficacious in reducing brain edema when administered 2–4 hours after the induction of ICH, and this beneficial effect remained for up to 24 hours postinjury. This finding is clinically relevant with respect to the fact that patients with stroke arrive at the hospital at a median of 4.3 hours after stroke onset[30]. Moreover, recovery of neurologic function occurs more rapidly when treatment with DFX is begun within 24 hours, suggesting greater efficacy with earlier treatment[31]. Additionally, the efficacy of DFX in animals in the present review was greatest at doses of 10–50 mg/kg (equivalent to 52.9–79.4 mg/kg in a 70-kg human), although no marked difference in efficacy was noted with the use of multiple doses. It should be noted that the optimal dose of DFX is species specific, and thus effective doses should be validated for each species accordingly. However, clinical data regarding the effectiveness and complications of DFX and other iron chelators in human patients with acute stroke, particularly in patients with ICH, are quite limited[9]. The results of a phase-I open-label study indicated that the maximum tolerated dose of DFX was 62 mg/kg per day (maximum of 6000 mg/day) and did not increase the incidence of serious adverse events[31]. Therefore, the clinically optimal dose of DFX may be 52.9–62.0 mg/kg. In most clinical trials involving humans, DFX was administered intravenously, which, though similar to intraperitoneal administration in animals, has a higher absorption rate and quicker absorption time. However, intramuscular injection of DFX showed significantly higher efficacy than intraperitoneal injection. The injection route of a given drug may have a significant impact on its effectiveness due to distinct biodistribution profiles. According to the plasma concentration–time curve of different drug delivery routes, the peak drug concentration after intraperitoneal injection is reached soon after the drug is administered. In one study, a DFX plasma concentration of 80–130 nmol/L was recorded 3 minutes after intravenous injection [9]. In contrast, the peak plasma concentration of DFX after intramuscular injection occurs several hours later, which is closer to the time point at which erythrocyte lysis occurs after ICH. Notably, however, intramuscular and intraperitoneal injections were difficult to compare in this review because in most of the included studies, DFX was administered multiple times. Another finding of the present study is that the efficacy of DFX was highest when ICH was induced under phenobarbital anesthesia. In one study, low-dose phenobarbital (30 mg/kg) showed a neuroprotective effect in mice with neonatal stroke[32]; in another study, phenobarbital augmented the neuroprotective efficacy of therapeutic hypothermia in hypoxic-ischemic encephalopathy[33]. These findings might confound the interpretation of the efficacy of DFX under the use of phenobarbital. Thus, identification of the optimal anesthetic or justification for rejecting another is not possible with the currently available data. With respect to the ICH induction method, the highest efficacy of DFX was found in studies that utilized the lysed erythrocyte model of ICH; high efficacy was also found in association with hemoglobin infusion. Hemolysis and subsequent hemoglobin toxicity occur 2–3 days after ICH, and brain edema peaks approximately 3–4 days after hemorrhage begins. Thus, brain edema, neurologic deficits, and other changes in lysed erythrocyte animal models of ICH occur 3–4 days earlier than in the two more frequently used animal models of ICH (the blood and collagenase infusion models)[34,35]. The recovery process may be initiated much earlier in animals with ICH induced by lysed erythrocyte infusion, possibly resulting in a better outcome. Therefore, repeating tests in multiple models is strongly suggested. Age is a critical factor affecting brain injury in both animals and humans with ICH[36], and it is encouraging that some of the included studies utilized aged animals, as the use of young animals in preclinical studies of ICH limits the direct translation into clinical trials[37]. Moreover, the effect size was higher in aged Fischer 344 rats (18 months of age, which corresponds to 50 years of age in humans)[13]. ICH led to more severe brain swelling and greater neurologic deficits in older than in younger rats. However, old and young rats exhibited identical temporal profiles of recovery[36], which resulted in a greater improvement during the recovery process in older rats (excluding the group in which age was not reported). This may explain the overestimation of the effect size in the aged group.Although studies involving aged animals were included in this systematic review, reports on certain types of comorbid animal models are still lacking, such as hypertensive and diabetic animal models. Case-control and cohort studies have provided abundant evidence of the fact that hypertension is the single most critical risk factor of ICH[38]. Diabetes is an additional proven risk factor for ICH and has gained increasing attention after the publication of a large meta-analysis in which the relative risk of ICH in patients with diabetes was found to be 1.6-fold that of patients without diabetes[39]. Thus, the predictive value of previous studies for clinical trials might be limited by this finding. Furthermore, the presence of comorbidities can affect the efficacy of various therapies in animal models. In order to exam whether the use of random and blind strategies influenced the effect size, we pooled ten studies from the same laboratory and then conducted a subgroup analysis. There was a higher effect size in studies that did not used blind assessment compared with those that did, which indicates that results may be confounded by an intended effect of the observers. And we also noticed that these ten studies from the same laboratory might cause bias, therefore, the results should be interpreted with caution.

### Limitations

The present study summarizes the available preclinical data on the therapeutic efficacy of DFX for ICH. However, the results should be interpreted with caution because of limitations to our approach. First, our analysis was only able to include available studies. Studies with negative findings are less likely to be published; thus, the present meta-analysis may have overstated the effect size. The trim-and-fill approach revealed 17 studies on neurobehavioral outcomes that were theoretically missing. Taking these theoretically missing studies into consideration, the effect size is likely to have been overstated. Second, whether the efficacy of drugs in studies involving animal models should be assessed by behavioral end points rather than volumetric, content-related, or other parameters has been previously debated[21]. However, behavioral outcomes are not accurate predictors of efficacy in clinical trials. In humans, the lesion volume and degree of brain edema are always determined by diffusion-weighted magnetic resonance imaging and are correlated with both clinical impairment and clinical outcomes. Additionally, observation of side effects in animal models is very difficult and represents a shortcoming of preclinical studies. Finally, we noticed that heterogeneity among the included studies was high with an *I* ^*2*^ value of 76.4%, which may have been caused by variability in the animal species, time windows, drug administration routes, drug doses, ICH induction methods, anesthetics, and observed end points among the included studies. Thus, whether the studies were adequately comparable for pooling in a meta-analysis is unclear. Control of heterogeneity is difficult; some degree of study heterogeneity due to factors such as the study design and methodological quality is expected, even with specific inclusion criteria that target the most homogeneous studies possible. Therefore, subgroup analysis was performed. Notably, the statistical power was limited because of an insufficient number of studies included in each subgroup. This may have led to insignificant discrepancy among subgroups due to underestimation. The present analysis may have thus failed to detect certain differences among the subgroups.Overall, the present review shows that DFX is a potentially effective neuroprotective treatment in animal models of ICH. Brain edema after ICH, which is mainly caused by iron overload, is considered to be one of the most devastating and life-threatening complications of ICH and is closely related to the development of neurologic deficits. DFX may represent a practical neuroprotective drug in clinical strategies against ICH because of its ability to target iron overload. Further confirmation is required by additional clinical studies. Therefore, bidirectional translational research between preclinical and clinical trials is critical. However, clinical evidence supporting or refuting the use of iron chelators in acute stroke treatment is not yet available; no clinical trials involving humans were found. Encouragingly, at least one such trial is ongoing[31].

## Supporting Information

S1 File Certification from Mejaden Biosience Company. (PDF)

S2 File PRISMA 2009 Checklist. (DOC)
