# Supplementary material for: Efficacy of Deferoxamine in Animal Models of Intracerebral Hemorrhage: A Systematic Review and Stratified Meta-Analysis
Source: PLoS One. 2015 May 22;10(5):e0127256. doi: 10.1371/journal.pone.0127256 (PMC4441464; doi:10.1371/journal.pone.0127256)
Supplement: S1 File — (PDF) [file pone.0127256.s001.pdf]

Certificate from *Medjaden* Bioscience Limited

Dear Sir or Madam:

This document certifies that the manuscript, **Efficacy of deferoxamine in animal models of intracerebral hemorrhage: a systematic review and stratified meta-analysis**, was edited for proper English language, grammar, punctuation, spelling, and overall style by highly qualified native English speaking and professional scientific at *Medjaden* Bioscience Limited on 22nd Dec. 2014. Neither the research content nor the authors' intentions were altered in any way during the editing process.

Yours sincerely,

(Miss) Selin Ho
for *Medjaden*
Medjaden Bioscience Limited
General Office
Room 2001-4, China Insurance Group Building,
141 Des Voeux Road, Central, Hong Kong
Tel: +852 81350971; Fax: +852 81350972
Email: medjaden@gmail.com
Website: www.medjaden.com

Information about *Medjaden Bioscience Limited*:
Medjaden is an emerging editing company that provides professional services for laboratory researchers, postgraduates, and clinical doctors. Our mission is to help our clients initiate, maximize and accelerate the production of articles from their research work. The company

employs experienced medical statisticians, writers, and editors drawn from both Chinese and English backgrounds worldwide, who strive to ensure that every edited article is published in an English journal, preferably one that is included in The Institute for Scientific Information (ISI) Citation Database, the Science Citation Index (SCI) Expanded, Index Medicus (IM), Medline, PubMed, EMBASE/Excepta Medica, and other scientific resources.
